# Supplementary material for: Structure and Activity of a Cytosolic Ribosome-Inactivating Protein from Rice
Source: Toxins (Basel). 2019 Jun 6;11(6):325. doi: 10.3390/toxins11060325 (PMC6628440; doi:10.3390/toxins11060325)
Supplement: Supplementary file 1 [file toxins-11-00325-s001.pdf]

Supplementary Materials: Structure and Activity of a Cytosolic Ribosome-Inactivating Protein from Rice

Jeroen De Zaeytijd, Pierre Rougé, Guy Smagghe and Els J.M. Van Damme *

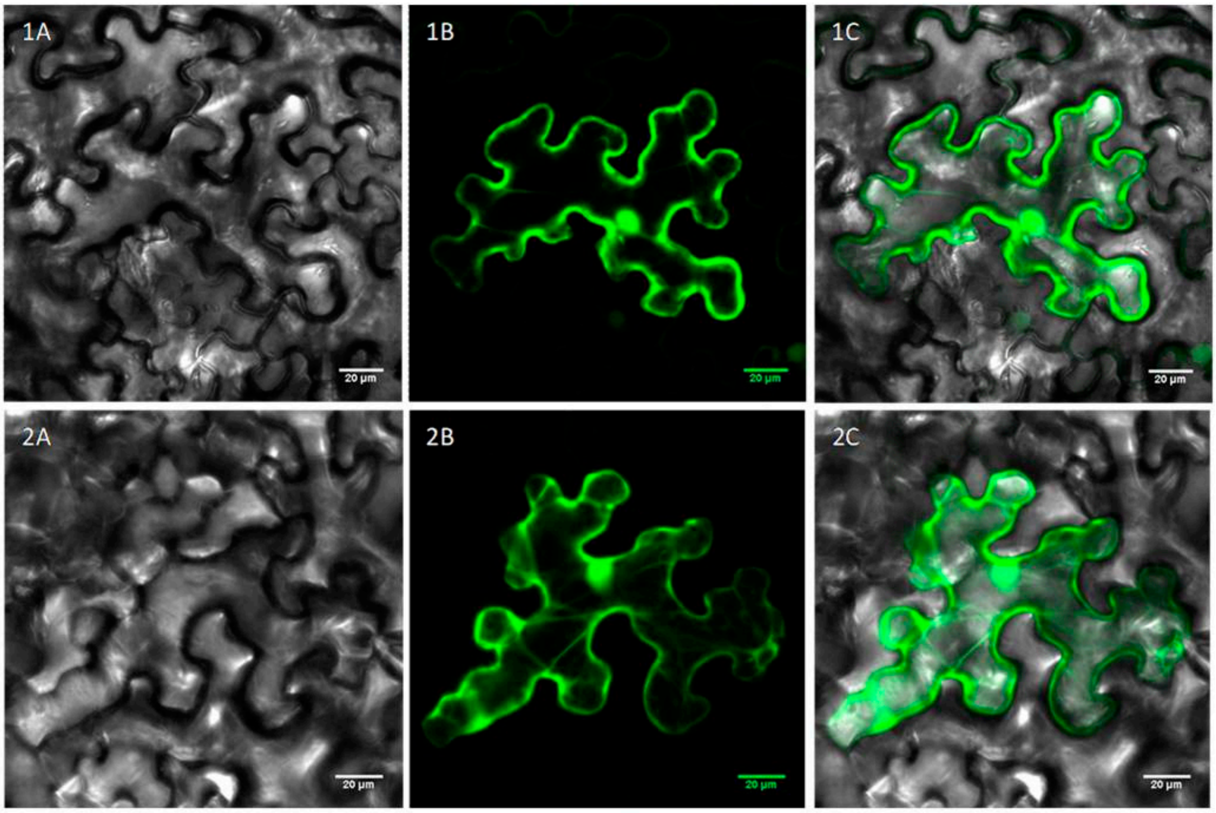

Figure S1. Subcellular localization of eGFP-OsRIP1 (1) and OsRIP1-eGFP (2) fusion proteins in *Nicotiana benthamiana* epidermal cells. Panels **1A** and **2A** show light transmission images of *Nicotiana benthamiana* epidermal cells. Panels **1B** and **2B** show eGFP fluorescence signals, panels **1C** and **2C** display transmission/fluorescence overlay pictures. Both eGFP-OsRIP1 and OsRIP1-eGFP fusion proteins display a similar fluorescent pattern, suggesting that the orientation of the eGFP tag did not influence the subcellular localization of the fusion proteins.

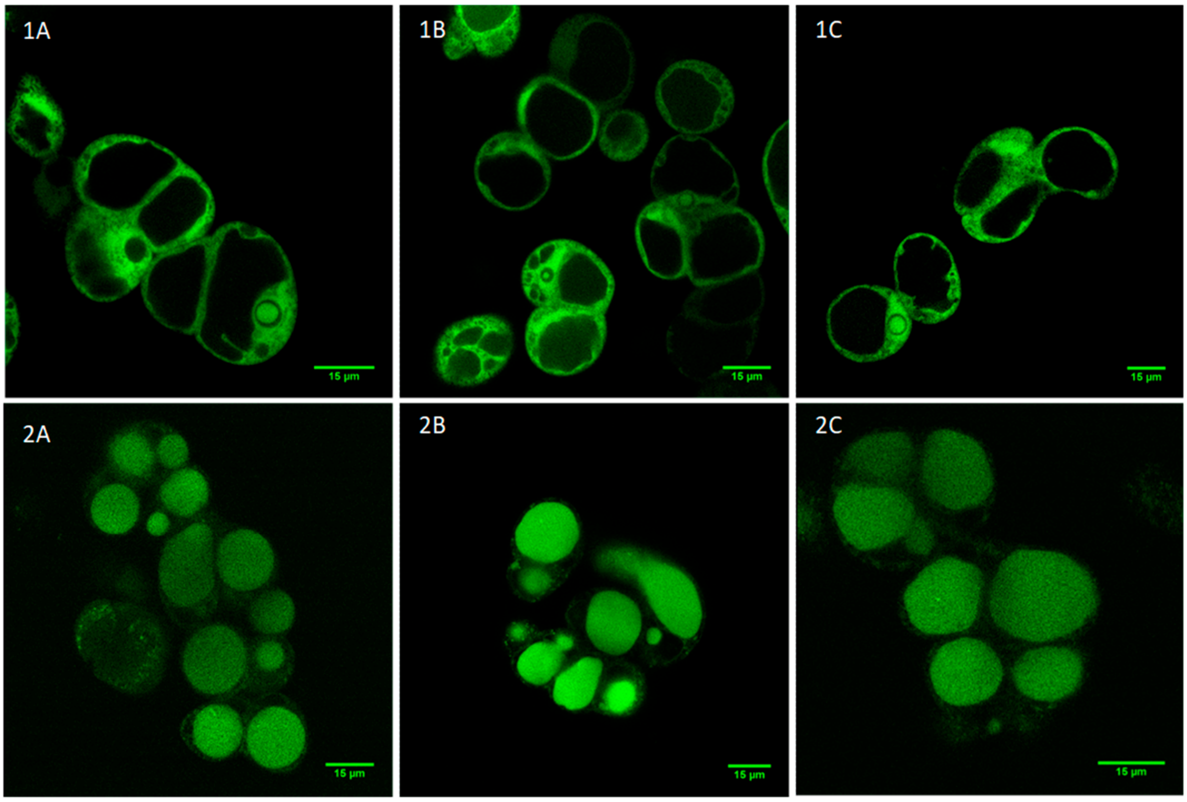

Figure S2. Subcellular localization of OsRIP1-eGFP (**1A–1C**) and SP-eGFP (**2A–2C**) fusion proteins in stably transformed PSB-D suspension cells. OsRIP1-eGFP fusion proteins are detected in the nucleocytoplasmic compartment while SP-eGFP fusion proteins end up in the vacuole.

Table 1. List of primers.

Primer	Sequence (5'-3')
P43	GTTCACAACGATCGAGCATC
P42	CTGTTCGATCAAGCAAGCTG
P44	ACCAGCAGCAGCAACTTTAG
EVD275	GCCTGAACACCATATCCATCC
EVD276	GCAGCTGAGAATATTGTAGGAGATC
P355	AAAAAGCAGGCTTCACCATGGCGTTGAACCCGCT
P356	AGAAAGCTGGGTGGTTCCCATGAAACAGCTGAAGC
P357	AGAAAGCTGGGTGTCAGTTCCCATGAAACAGCTGAAGC
EVD2	GGGGACAAGTTTGTACAAAAAAGCAGGCT
EVD4	GGGGACCACTTTGTACAAGAAAGCTGGGT
EVD386	GTAAAACGACGGCCAG
EVD387	CAGGAAACAGCTATGAC
EVD472	GAAACCTCCTCGGATTCCAT
P1	AGGTCACTGGATTTTGGTTT
P358	ATGATGATGATGTCCTCCTCCGTTCCCATGAAACAGCTGA
P359	TTAAGAAGGAGATATACGGGATGGCGTTGAACCCGCT
P216	GCTTTGTTAGCAGCCGGATCTCAATGATGATGATGATGATGTCC
P242	GATCCGGCTGCTAACAAAG
P243	CCCGTATATCTCCTTCTTAAAG
